# Epicardial fat and insulin resistance in healthy older adults: a cross-sectional analysis

**DOI:** 10.1007/s11357-023-00972-6

**Published:** 2023-10-19

**Authors:** Aliki Kalmpourtzidou, Ilaria Di Napoli, Alessandra Vincenti, Rachele De Giuseppe, Pietro Mariano Casali, Chiara Elena Tomasinelli, Fulvio Ferrara, Francesco Tursi, Hellas Cena

**Affiliations:** 1 Laboratory of Dietetics and Clinical Nutrition, Department of Public Health, Experimental and Forensic Medicine, University of Pavia, 27100 Pavia, Italy; 2 Department of Public Health, Experimental and Forensic Medicine, University of Pavia, Pavia, Italy; 3 Laboratory Medicine Department - Centro Diagnostico Italiano, Milan, Italy; 4 Complife Italia s.r.l., Piazzale Siena 11, 20146 Milano, Italy; 5 Clinical Nutrition and Dietetic Service, Unit of Internal Medicine and Endocrinology, ICS Maugeri IRCCS, 27100 Pavia, Italy

**Keywords:** Epicardial fat, Insulin resistance, Free-living older adults, HOMA-IR index

## Abstract

Insulin resistance (IR) and cardiovascular diseases (CVD) are relevant concerns in the elderly population; as the world’s population ages, IR and CVD are two universal public health problems. While a link between IR a CVD has been established, the mediating mechanisms are uncertain and rigorous investigations are needed to fully elucidate them. The study aimed at assessing the relationship between epicardial fat (EF), an indicator of cardiovascular risk, and IR in Italian free-living elderly (*n* = 89). Baseline data from a previous cohort was used. Anthropometric measurements, EF, and IR-related variables, including the HOMA-IR index and other biochemical parameters were obtained. The correlation between EF and IR was explored. Further analysis was conducted to identify significant differences regarding IR variables among EF quartiles. EF correlated positively with glucose levels in females, males and the total population. The pairwise comparison among EF quartiles showed significant differences in glucose levels, HOMA-IR index, triglycerides, and total cholesterol levels. To our knowledge, this is the only study assessing the relationship between EF and IR in healthy elderly, while most of the studies have investigated EF and IR in diseased populations. Further research with a longitudinal approach should be conducted to design concrete conclusions about this relationship.

## Introduction

The definition of elderly varies and ranges from 50 to 80 years and above; in medical research usually elderly are defined as populations above 65 years [1]. Nowadays in many countries, most people could live at least 60 years due to lower mortality at a young age in low- and middle-income countries and increased quality of life in high-income countries [2]. World Health Organization (WHO) published the “Global Strategy and action plan on ageing and health” in 2016 to highlight the actions that are needed for a long and healthy life for everyone worldwide including tackling Non-Communicable Diseases (NCDs) [3, 4] which can be also prevented by adopting a correct lifestyle and maintaining an adequate nutritional status [3, 4].

However, all age-related changes are accompanied by a decline in biological function due to a low-grade chronic inflammatory state (also named inflammaging) and increased vulnerability leading to frailty [5]. Advanced age is thus identified among the major risk factors of the main NCDs, including also cardiometabolic diseases which are the major cause of death in older age [2, 5]. Concerning cardiovascular diseases (CVDs), the risk is increased in the elderly irrelevantly of the sex [5]; multiple risk factors such as hypertension, diabetes, and obesity have been associated with CVDs [6–9].

Ageing is also associated with changes in body fat mass and particularly, epicardial fat (EF) thickness is an indicator of metabolic and cardiovascular risk [10]; it has been reported that EF is thicker in older adults and consequently, there is a higher risk for CVDs [10–12]. Indeed, EF is a visceral fat deposit, located between the heart and the pericardium and accounting for approximately 20% of the total heart weight [10]. Physiologically, EF plays a cardio-protective role by providing mechanical protection, serving as an energy source to the myocardium as well as producing anti-inflammatory adipokines [13]. Indeed, EF produces and secretes numerous bioactive molecules (secretome) which are then transported into the adjacent myocardium via vasocrine and/or paracrine pathways [14]. Given the lack of anatomical barriers separating the two tissues, the molecules secreted by the EF pour directly into the myocardium and coronary lumen [13]. Thus EF can be considered an endocrine organ with local effects on the heart [13]. Finally, it is also hypothesized that EF functions as brown adipose tissue generating heat in response to low temperatures and activating the autonomic nervous system [13].

However, when changes occur in the local microenvironment, the positive and beneficial effects of EF can turn into harmful effects [15]. This transition occurs in various pathological conditions, including cardiometabolic diseases, favouring the shift of the EF phenotype and secretome towards a pro-inflammatory, pro-fibrotic and pro-atherosclerotic profile [15]. Thus, EF shares many of the pathophysiological properties of other visceral fat deposits [15] since it releases many of the pro-atherogenic and pro-inflammatory cytokines also produced by the visceral abdominal fat (e.g. monocyte chemotactic protein-1, tumor necrosis factor, in-terleukin-6, resistin, and several adipokines) [15].

Older adults are more glucose intolerant and insulin resistant than the younger ones having a higher risk of developing type 2 diabetes mellitus (T2DM) [16, 17]. It is well-recognized that exercise and/or weight loss could increase glucose disposal and as a result in insulin sensitivity in older adults [16–20]. Insulin resistance (IR) tests are a better predictor of CVDs risk than solely fasting glucose levels [18].

Multiple studies have associated IR with cardiovascular risk with or without the existence of other CVDs factors or diabetes [19–26]; EF is also associated with other known factors, such as obesity, diabetes mellitus, age and hypertension, which interprets its role as an independent risk marker intricate.

However, available studies about the link between EF and IR were mainly conducted in patients with diabetes and/or obesity and/or CVDs. For instance, according to Iacobellis et al. [27], EF and obesity-related insulin resistance were significantly correlated in middle-aged adults. A systematic review and meta-analysis by Li et al. [28] reported that EF thickness was significantly higher in diabetic patients than in nondiabetic patients. Moreover, Güneş et al. [29] conducted a study on children with obesity and noticed a positive correlation between EF and insulin resistance. Finally, Baldasseroni et al. [30] suggested that among patients with coronary artery disease, the ones with diabetes and a history of hypercholesterolemia had a higher EF compared to controls.

The evidence of studies mentioned above shows a relation between CVDs and IR, however, there is a lack of evidence about a potential association. Few studies are available about the relationship between EF and IR in healthy populations and specifically, even though it is considered a reliable CVD risk indicator. Therefore, this study may enhance insights into the potential correlation between EF IR-related variables in older healthy and free-living adults.

## Materials and methods

### Study population

Baseline data from the Food Social Sensor Network (FoodNet) project [31] were used. The FoodNet research, a prospective cohort study that lasted for 3 years from 2017 to 2021, aimed at meeting the nutritional needs of the free-elderly living in Milan (Italy) aged ≥ 65 years, by providing new food products more suited to the specific needs of consumers, as well as short- and long-term food policies to reduce their risk of food insecurity in the elderly [31]. The FoodNET project was funded by REGIONE LOMBARDIA—Research and Innovation, co-funded by POR FESR 2014-2020 (CUP E47F17000020009) and was previously described elsewhere [31]. The study was conducted under the Declaration of Helsinki and approved by the Institutional Review Board approved by the Ethics Committee of the University of Milano-Bicocca (protocol 410LABRA, 09/21/2018). Informed consent was obtained from all subjects involved in the study.

The present research consisted of a nested cross-sectional analysis of data from the baseline of the cohort. Hence, out of 437 participants at baseline, 89 (20%) attended the Complife Italia s.r.l. (Piazzale Siena 11, 20146 Milano, Italy; https://complifegroup.com) from June 2019 and March 2021, were included, according to the following inclusion criteria: (i) aged ≥ 65 years; (ii) free-living; (iii) not on long-term regular vitamin/omega-3 polyunsaturated fatty acids (e.g. eicosapentaenoic acid, EPA; docosahexaenoic acid, DHA) supplementation; (iv) not affected by hepatic and renal diseases; bleeding disorders; mental and neurological disorders; severe eating disorders; and exclusion criteria i) institutionalized; (iii) the presence of certain diseases, including hepatic and renal diseases; bleeding disorders; mental and neurological disorders; severe eating disorders; iv) subjects on long-term regular vitamin/EPA or DHA supplementation.

Medical history was taken from the participants for the identification of chronic morbidities, such as T2DM, osteoporosis, hypercholesterolemia, arthritis, and potential pharmacological treatment.

Participants were also evaluated for (i) anthropometric measurements (height; weight; waist circumference, WC); (ii) body composition and phase angle (PhA); (iii) muscle strength; (iv) biochemical parameters such as serum glucose and insulin levels; glycated haemoglobin levels (HbA1c); lipid panel parameters (serum levels of total cholesterol, TC; triglycerides, TG; HDL-cholesterol, HDL; LDL-cholesterol, LDL); serum 25-hydroxyvitamin D levels, 25-OH-D; total plasma homocysteine levels (tHcy) and serum metabolically related vitamins (vitamin B_12_ levels, B_12_ and folate levels, Fol). The Body Mass Index (BMI) and homeostasis model of insulin resistance (HOMA-IR) were calculated [32, 33].

As sarcopenia, is a common age-related condition that can significantly impact the health and well-being of older adults, the presence or risk of sarcopenia has also been described by evaluating the appendicular skeletal muscle mass (ASMM) and muscle strength was also taken into account, respectively, according to the European Working Group on Sarcopenia in Older People (EWGSOP2) Consensus [34].

### Echocardiographic assessment of epicardial fat thickness

Based on Iacobellis et al. [35], EF thickness was measured through an echocardiographic assessment by trained personnel. Participants laid at the left lateral decubitus position and EF thickness was measured on the free wall of the right ventricle in two-dimensional long and short heart axis views, at the end of the systole, using Vivid iq GE Healthcare (Milan, Italy) with a 3.5-–7.5-MHz variable-frequency transducer. According to the literature, the elderly has 20–25% ticker EF than younger adults; as a result, 8 mm was defined as the cut-off for healthy free-living elderly for this analysis. [5, 11, 12, 35–37]. Based on this cutoff, in the second part of the statistical analysis, participants were divided into quartiles according to their EF measurements: Q1: ≤ 8 mm, Q2: 8.1–10 mm, Q3: 10.1–12 mm and Q4: > 12 mm.

### Anthropometric measurements, body composition, phase angle, and muscle strength

Height (cm), weight (kg) and WC (cm) were measured with the use of standardized procedures [32]. In brief, WC (cm) was measured to the nearest centimeter with a flexible steel tape measure with participants standing, arms crossed, and placing hands on opposite shoulders [32]. After exhaling smoothly, abdominal WC was measured in the horizontal plane between the lowest portion of the rib cage and the superior lateral border of the right ilium [32]. The BMI was then calculated, as the ratio between weight and height squared with weight in kilograms and height in meters [32].

Body composition and PhA were evaluated with the use of single frequency (50 kHz) bioelectrical impedance analysis (BIA; BIA-101 model; Akern, Florence, Italy), with an alternating electric current at low intensity (800 μA) [38]. Briefly, BIA measures resistance and reactance; after including age, (years), sex, height (cm) and weight (kg), the predictive equations estimate fat mass (FM, indicating the body component without water), fat-free mass (FFM, representing the remaining body components: skeletal muscle tissue, internal organs, interstitial fat), body cell mass (BCM, reflecting metabolically active tissue and intracellular water) and PhA (proportional to the ratio between reactance, related to FFM, and resistance, dependent on BCM) [39]. The normal PhA value for adults is expressed in degrees and the relevant normal reference interval ranges from 5° to 7° [39].

The appendicular skeletal muscle mass (ASMM) was calculated as the sum of the arm and leg lean mass (kg/height (m^2^) [34].

Muscle strength was measured with the use of a hand-grip dynamometer (DynEX, Akern srl), according to standardized procedures [39]. Briefly, hand grip strength was assessed in the standing position, with the dominant arm held away from the body, and exerting maximal hand contraction strength [39]. The test was repeated three times; muscle strength was determined considering the mean value of the measurement.

Finally, a diagnosis of sarcopenia was provided [34]. Precisely, when ASMM was below the cut-off of 20 kg for men and 15 kg for women and the handgrip strength was below the cutoff of 27 kg for men and 16 kg for women, sarcopenia was defined, according to the EWGSOP2 Consensus [34, 40–42].

### Biomarkers analysis

Biochemical parameters were evaluated in the context of the assessment of the nutritional status of the subjects enrolled in the study. In particular, blood samples were drawn in the morning, after an overnight fast. For the analysis of the biochemical parameters, 4 blood specimens from each subject were collected in tubes, either with no additive (2 specimens, for serum lipid parameters, B_12_, Fol, glucose and insulin levels assessment) or containing EDTA (2 specimens) to prevent coagulation.

One EDTA specimen was used to assess HbA1c while the other one was centrifuged to obtain plasma samples (for tHcy and 25-OH-D levels). All the biochemical parameters were measured at the Centro Diagnostico Italiano (CDI) S.p.A (via Simone Saint Bon 20, 20147, Milano, Italy) by routine methods.

Serum lipid parameters levels (TC, TG, HDL, LDL), vitamins levels (B_12_ and Fol), glucose, and insulin levels as well as plasma 25-OH-D and Hcy levels were measured on Atellica ® CH Analyzer (Siemens Healthcare Diagnostics Inc., 511 Benedict Avenue, Tarrytown, NY, USA) with the relevant commercial reagent kits. According to the manufacturer, the linearity through the measurement range is between 10 and 550 mg/dL (0.11–6.22 mmol/L) for TG; 20–129 mg/dL (0.52–3.34 mmol/L) for HDL; 5.0–1000.0 mg/dL (0.13–25.90 mmol/L) for LDL; 25–618 mg/dL (0.65–16.01 mmol/L) for TC.

The limit of blank (LoB), the limit of detection (LoD), and the limit of quantitation (LoQ) are 3 mg/dL (0.03 mmol/L), 10 mg/dL (0.11 mmol/L), and 4 mg/dL (0.05 mmol/L) for TG, respectively; 0.5 mg/dL (0.01 mmol/L), 2.7 mg/dL (0.07 mmol/L), and 2.1 mg/dL (0.05 mmol/L) for HDL, respectively; 1 mg/dL (0.03 mmol/L), 25 mg/dL (0.65 mmol/L), and 2 mg/dL (0.04 mmol/L) for TC, respectively; 0.6 microU/mL, 0.8 microU/mL, 0.5–300.0 microU/mL for insulin, respectively; 1.70 ng/mL (4.25 nmol/L), 3.20 ng/mL (8.00 nmol/L), 4.20 ng/mL (10.50 nmol/L) for 25-OH-D, respectively; an LoB of 0.8 mg/dL (0.02 mmol/L) and an LoD of 3.0 mg/dL (0.08 mmol/L) for LDL; an LoB of 45 pg/mL (33 pmol/L), an LoD of 90 pg/mL (66 pmol/L) for B12; an LoB of 0.40 ng/mL (0.91 nmol/L) and an LoD of 0.70 ng/mL (1.59 nmol/L) for Fol; an LoB of 0 mg/dL (0.0 mmol/L) and an LoD of 1 mg/dL (0.1 mmol/L) for glucose; an LoB of 0.50 μmol/L and an LoD of 0.67 μmol/L for Hcy.

HbA1c (expressed as % or mmol/mol) was assessed by the ion-exchange HPLC method that was performed on the Tosoh Automated Glycohemoglobin Analyzer HLC-723G11 Variant Analysis Mode (HLC-723 G11 apparatus, Tosoh Bioscience, Inc., Tokyo, Japan), as previously described [43].

Homeostasis model assessment of insulin resistance (HOMA-IR) was then calculated to measure IR, by using the following equation [44]:

$$\mathrm{fasting}\;\mathrm{insulin}\;(\mathrm{microU}/\mathrm{mL})\;\mathrm x\;\mathrm{fasting}\;\mathrm{glucose}\;(\mathrm{nmol}/\mathrm L)/22.5$$

Insulin resistance was defined for HOMA-IR index (HOMA-IR) ≥ 2.5 [33, 44].

Hyperhomocisteinemia (HHcy), an independent factor for CVDs [45], was defined for tHcy levels at or above 15 μmol/L [45]. HHcy was also classified as mild (15–30 μmol/L), moderate (30–100 μmol/L) and severe (> 100 μmol/L) [45].

### Statistical analysis

Categorical variables were presented as numbers and percentages. Continues variables were presented mainly as medians and interquartile ranges (IQRs) due to the non-normal distribution followed by most of the variables. Q-Q plots and the Kolmogorov-Smirnof test were used for the normality assessment of the variables.

The Spearman’s correlation coefficient was used to correlate EF, glucose, insulin, HbA1c and HOMA-IR index among them and other baseline variables for the male, female and total population. Non-parametric Kruskal-Wallis test with pairwise comparisons was performed to assess the differences among EF quartiles defined above regarding age, WC, BMI, glucose, insulin, HOMA-IR index, total cholesterol, and triglycerides levels. The level of significance was set at *p*-value < 0.05. Statistical analysis was performed using the software IBM SPSS v. 28.

## Results

### General characteristics of the population

Eighty-nine free-living older adults previously enrolled in the FoodNet project [31] were considered. The mean age of the population was 71 (range 64–84 years) and most of the participants were women (71%). Most of the older adults (82.0%) had a WC that indicated a risk for CVDs (≥ 80 cm for women and ≥ 94 cm for men) [46], while 26.5% of them had obesity (BMI ≥ 30 kg/m^2^). Eighty-eight percent of subjects were measured with an EF above the CVDs-related risk cut-off (≥ 8 mm) [10, 11, 36, 37] for the elderly. Insulin resistance was reported for 16.9% of subjects and HbA1c levels (cut-off ≥ 42 mmol/mol) [47] were impaired in 13.5% of the sample. High total cholesterol levels (cut-off ≥ 200 mg/dl) [48, 49] were observed for 49.4% and high triglyceride levels (cut-off ≥ 150 mg/dl) [49] for 13.5% of the older adults. Vitamin D deficiency (25(OH)D plasma levels < 30 ng/ml) [50] was common and observed in 64.0% of participants (Table 1).

**Table 1:** General characteristics of the FoodNet participants

	*N* total	Measurement unit	Median (IQR)	Cutoff	*n* based on cutoff (%)
BMI	83	kg/m^2^	25.9 (23.6–30.1)	For obesity ≥ 30 kg/ m^2^	22 (26.5)
WC	89	cm	93 (86–101)	CVD risk for women: ≥ 80 cm [46] CVD risk for men: ≥ 94 cm [46]	73 (8.02)
Glucose	89	mg/dL	88 (83–97)	90–150 mg/dL [76, 77]	< 90 mg/dL: 83 (93.3) > 150 mg/dL: 3 (3.4)
Insulin	89	microU/mL	5.7 (3.8–8.6)	4.2–11.7 microU/mL [33]	< 4.2 microU/mL: 26 (29.2) > 11.7 microU/mL: 9 (10.1)
HbA1c	89	mmol/mol	37 (35.5–39)	Risk for diabetes: ≥ 42 mmol/mol [47]	12 (13.5)
HOMA-IR index	89		1.3 (0.8–2.1)	> 2.5 [78, 79]	15 (16.9)
ASMM	71	kg	16.8 (15–20)	Sarcopenia < 20 kg men < 15 kg women [40]	9 (12.7)
Phase angle	71	°	5.3 (5–5.9)	Normal range 5–7° [80]	< 5°: 4 (5.6)* > 7°: 1 (1.4)*
Handgrip strength	67	kg	20.1 (16.9–24.1)	Risk for sarcopenia < 27 kg men < 16 kg women [42]	16 (23.9)
EF	89	mm	10.8 (9.3–12.5)	Risk for CVDs ≥ 8 mm [10, 11, 36, 37]	78 (87.6)
TC	89	mg/dl	199 (178–217)	High levels ≥ 200 mg/dl [48, 49]	44 (49.4)
TG	89	mg/dl	94 (71.5–126)	High levels ≥ 150 mg/dl [49]	12 (13.5)
HDL	89	mg/dL	61 (50–73)	Low levels < 40 [49]	6 (6.7)
LDL	89	mg/dL	130 (109–152)	High levels ≥ 130 mg/dl [56]	46 (40.9)
Vitamin B12	89	pg/mL	379 (326–468)	B_12_ deficiency < 150 pmol/L (203 pg/mL) for serum vitamin B_12_ [51]	1 (1.1)
Folate	89	ng/mL	9.7 (6.8–13.2)	Folate deficiency < 4.4 ng/mL [51]	9 (10.1)
tHcy	89	μmol	13.3 (11.1–15.7)	Mild HHcy: 15–30 μmol/L Moderate HHcy: 30–100 μmol/L Severe HHcy: > 100 μmol/L [45]	24 (27.0) 3 (3.4) 0(0)
25(OH)D	89	ng/ml	27(19.5–33.5)	25(OH)D deficiency < 30 ng/ml [50]	57 (64.0)

Regarding homocysteine metabolism, plasma tHcy levels were within the relevant reference interval (5–15 μmol/L) [45] in almost all the participants (69.7%), as well as the levels of Hcy metabolically related vitamins. Indeed, only 30.0% of the older adults reported tHcy levels above the cut-off value and serum levels of B_12_ and Fol were below the cut-off values (< 150 pmol/L for B_12_ and < 4.4 ng/mL for Fol) [51] only in 1.1% and in 11.2% of participants, respectively. Again, sarcopenia (based on ASMM measurement) [34] was reported only in 12.7% of participants while 23.9% of subjects were at risk of developing sarcopenia (based on handgrip strength measurement; cut-off values: < 27 kg in men and < 16 kg in women) [42].

Last, phase angle, a proxy for the assessment of nutritional status (normal reference interval: 5°–7°) [38] was within the relevant reference interval in most of the study population (93.0%). Thus, since almost all the subjects had good nutritional status and did not have sarcopenia or hyperhomocysteinemia, these outcomes were not considered in the subsequent analyses.

### The correlations among EF and insulin resistance-related variables

The Spearman’s correlation coefficients (Table 2) showed that EF was positively correlated with glucose levels and 25(OH)D levels (*p*-value < 0.001) for females and total, but not in the male population. Glucose levels positively correlated with TG levels in male, female and total population while correlated negatively both with HDL levels (male and total population) and 25(OH)D levels (female and total population). Moderate positive correlations were found between insulin and BMI, WC, and TG for the male, female, and total population. Insulin levels were also weakly and positively correlated to ASMM in the female population while a negative correlation was described between insulin and HDL levels in male, female, and total population. Also, the HOMA-IR index was moderately and positively correlated with BMI, WC and TG in male, female and total population as well as correlated weakly with ASMM in the female population. A negative correlation was described between the HOMA-IR index and HDL levels in males, females and the total population. Weak positive correlations were noticed between BMI, WC, ASMM, and HbA1c levels in the female population while positive correlation was reported between TG and HbA1c levels in the female and total population. HbA1c levels correlated negatively with HDL levels only in the female population.

**Table 2:** The correlations among EF and insulin resistance-related variables for the male, female, and total sample population expressed as correlation coefficient (*p*-value)

	EF			Glucose levels			Insulin levels			HOMA-IR index			HbA1c		
	Male	Female	Total	Male	Female	Total	Male	Female	Total	Male	Female	Total	Male	Female	Total
Age	− 0.75 (0.716)	− 0.187 (0.142)	− 0.132 (0.216)	0.078 (0.706)	− 0.290 **(0.021)**	− 0.147 (0.170)	− 0.129 (0.532)	0.107 (0.402)	0.058 (0.587)	− 0.127 (0.536)	0.244 (0.054)	0.031 (0.774)	− 0.367 (0.065)	− 0.063 (0.624)	− 0.154 (0.149)
BMI	− 0.031 (0.884)	0.178 (0.211)	0.152 (0.168)	0.284 (0.168)	0.181 (0.175)	0.209 (0.058)	**0.517 (0.008)**	**0.453 (< 0.001)**	**0.463 (< 0.001)**	0.438 **(0.029)**	**0.432 (< 0.001)**	**0.428 (< 0.001)**	0.158 (0.452)	0.297 **(< 0.024)**	0.206 (0.061)
WC	0.112 (0.586)	− 0.067 (0.601)	− 0.012 (0.913)	0.273 (0.177)	0.057 (0.659)	0.153 (0.153)	0.433 **(0.027)**	**0.430 (< 0.001)**	**0.426 (< 0.001)**	0.373 (0.061)	**0.395 (0.001)**	**0.397 (< 0.001)**	− 0.016 (0.937)	**0.331 (0.008)**	0.146 (0.171)
EF	−	−	−	0.343 (0.086)	**0.454 (< 0.001)**	**0.442 (< 0.001)**	0.040 (0.845)	0.143 (0.263)	0.135 (0.206)	0.077 (0.709)	0.226 (0.075)	0.204 (0.55)	− 0.035 (0.865)	− 0.095 (0.457)	0.051 (0.638)
Glucose levels	0.343 (0.086)	**0.454 (< 0.001)**	**0.442 (< 0.001)**	−	−	−	**0.622 (< 0.001)**	**0.413 (< 0.001)**	**0.487 (< 0.001)**	**0.793 (< 0.001)**	**0.588 (< 0.001)**	**0.655 (< 0.001)**	0.264 (0.193)	**0.477 (< 0.001)**	**0.386 (< 0.001)**
Insulin levels	0.040 (0.845)	0.143 (0.263)	0.135 (0.206)	**0.622 (< 0.001)**	**0.413 (< 0.001)**	**0.487 (< 0.001)**	−	−	−	**0.947 (< 0.001)**	**0.971 (< 0.001)**	**0.966 (< 0.001)**	0.035 (0.866)	**0.386 (0.002)**	0.263** (0.013)**
HOMA-IR index	0.077 (0.709)	0.226 (0.075)	0.204 (0.55)	**0.793 (< 0.001)**	**0.588 (< 0.001)**	**0.655 (< 0.001)**	**0.947 (< 0.001)**	**0.971 (< 0.001)**	**0.966 (< 0.001)**	−	−	−	0.185 (0.366)	**0.464** **(< 0.001)**	**0.356 (< 0.001)**
Handgrip	− 0.155 (0.650)	0.209 (0.122)	0.059 (0.638)	− 0.155 (0.649)	0.159 (0.241)	0.163 (0.188)	− 0.336 (0.312)	0.129 (0.344)	0.081 (0.516)	− 0.73 (0.832)	0.146 (0.284)	0.137 (0.270)	− 0.110 (0.748)	0.182 (0.178)	0.116 (0.352)
ASMM	0.010 (0.961)	− 0.27 (0.858)	− 0.054 (0.653)	0.082 (0.702)	0.153 (0.306)	0.176 (0.143)	0.085 (0.692)	0.303 **(0.038)**	0.176 (0.143)	0.054 (0.802)	0.334 **(0.022)**	0.208 (0.082)	− 0.290 (0.169)	0.339 **(0.020)**	− 0.55 (0.650)
TC	− 0.121 (0.555)	0.193 (0.129)	0.100 (0.350)	− 0.263 (0.194)	0.212 (0.096)	0.011 (0.919)	− 0.298 (0.139)	0.059 (0.646)	− 0.079 (0.463)	− 0.316 (0.115)	0.123 (0.337)	− 0.061 (0.569)	0.034 (0.869)	0.057 (0.656)	0.110 (0.303)
TG	0.251 (0.217)	0.051 (0.692)	0.095 (0.374)	**0.465 (0.017)**	**0.365 (0.003)**	**0.372 (< 0.001)**	**0.591 (0.001)**	**0.526 (< 0.001)**	**0.530 (< 0.001)**	**0.567 (0.003)**	**0.565 (< 0.001)**	**0.545 (< 0.001)**	0.221 (0.277)	**0.283 (0.024)**	**0.279 (0.008)**
HDL	− 0.246 (0.226)	− 0.015 (0.905)	− 0.106 (0.324)	− **0.404 (0.041)**	− 0.242 (0.056)	− **0.339 (0.001)**	− **0.655 (< 0.001)**	− **0.561 (< 0.001)**	− **0.586 (< 0.001)**	− **0.620 (< 0.001)**	− **0.546 (< 0.001)**	− **0.572 (< 0.001)**	0.115 (0.576)	− **0.395 (0.001)**	− 0.173 (0.105)
LDL	− 0.124 (0.547)	0.065 (0.615)	− 0.005 (0.964)	− 0.215 (0.292)	0.176 (0.167)	0.012 (0.908)	− 0.188 (0.359)	0.193 (0.129)	0.060 (0.574)	− 0.217 (0.288)	0.232 (0.068)	0.056 (0.603)	− 0.050 (0.809)	0.182 (0.153)	0.139 (0.195)
25(OH)D	− 0.011 (0.958)	− **0.436 (< 0.001)**	− **0.351 (< 0.001)**	0.003 (0.987)	− **0.365 (0.003)**	− **0.292 (0.005)**	− 0.251 (0.217)	− 0.011 (0.931)	− 0.084 (0.432)	− 0.190 (0.352)	− 0.098 (0.447)	− 0.132 (0.219)	0.089 (0.664)	− 0.088 (0.490)	− 0.016 (0.882)

Significant *p*-value < 0.05, *P*-values of statistically significant correlations are highlighted in bold numbers when *p*-value < 0.05. Correlation coefficients and *p*-values of statistically significant correlations are highlighted in bold numbers when *p*-value < 0.01

### General characteristics of the population based on quartiles division

The pairwise comparison analysis conducted per EF groups using the Kruskal-Wallis test showed that significant differences were noticed mainly between the first three groups and the fourth one with a range of EF above 12 mm. Glucose levels differed significantly between Q2 and Q4 (*p* < 0.001), Q1 and Q4 (*p* = 0.002), and Q3 and Q4 (*p* = 0.006) quartiles (Tables 3 and 4). Specifically, glucose levels were the highest (96 mg/dl) in Q4 (Table 3). Insulin levels differed significantly only between Q3 and Q4 quartiles (*p* = 0.013) (Table 4), being higher in Q4 (microU/mL) compared to Q1 (5.7 microU/mL) (Table 3). HOMA-IR index scores differed significantly between Q1 and Q4 (*p* = 0.021) and Q3 and Q4 (*p* = 0.013) quartiles (Table 4). HOMA-IR index score was the highest (1.9) in comparison to the other quartiles (Table 3). Triglycerides levels differed significantly only between Q3 and Q4 quartiles (*p* = 0.029), being higher in Q4 (111 mg/dl) compared to Q3 (83 mg/dl) (Table 4 and Table 3, respectively). Total cholesterol levels differed significantly between Q1 and Q3 (*p* = 0.023) and Q3 and Q4 (*p* = 0.004), with Q1 (209 mg/dl) and Q4 (209 mg/dl) having the highest total cholesterol levels (Table 4 and Table 3, respectively). Last, significant differences were found among 25(OH)D levels and EF quartiles (Table 4); indeed, vitamin levels differed significantly between Q1 (30 ng/mL) and Q3 (28 ng/mL) as well as between Q3 (28 ng/mL) and Q4 (23 ng/mL) quartiles (Table 3).

**Table 3:** General characteristics of the population based on EF quartiles division expressed as medians (IQRs)

EF	≤ 8 mm (Q1)*	8.1–10 mm (Q2)	10.1–12 mm (Q3)	> 12 mm (Q4)
*N*	11	25	26	27
*n* female (%)	10 (91)	18 (72)	16 (62)	19 (70)
*n* pharmacological treatment (%)	1 (17)	7 (29)	8 (31)	15 (56)
Age (year)	70 (67–73)	72 (68–77)	71 (68–74)	68 (66–74)
BMI (kg/m2)	25 (23–29)	26 (24–28)	26 (23–28)	29 (25–32)
WC (cm)				
Men	104 (104–104)	94 (87–100)	92 (87–98)	95 (87–105)
Women	92 (86–100)	95 (87–100)	92 (86–100)	95 (85–105)
Glucose (mg/dl)	85 (79–89)	85 (79–89)	88 (82–97)	96 (91–112)
Insulin (microU/mL)	5.7 (2.9–7.2)	6 (3.1–9.3)	5.1 (3.6–6.3)	7.2 (4.7–12.4)
HOMA-IR index	1.1 (0.6–1.5)	1.3 (0.6–2.2)	1.2 (0.8–1.8)	1.9 (1.1–3.1)
HbA1c (mmol/mol)	38 (35–40)	36 (35–39)	37 (36–39)	37 (36–40)
TC (mg/dl)	209 (179–255)	199 (178–217)	184 (163–207)	209 (197–239)
TG (mg/dl)	86 (57–160)	95 (81–111)	83 (67–112)	111 (72–153)
HDL (mg/dL)	69 (58–82)	61 (49–69)	62 (52–72)	57 (49–70)
LDL (mg/dL)	135 (109–150)	142 (117–153)	118 (95–144)	130 (112–165)
25(OH)D(ng/ml)	30 (27–44)	28 (22–35)	28 (17–34)	23 (14–28)

*EF cutoff for healthy free-living older adults

**Table 4:** Differences and pairwise comparisons among EF quartiles using independent-samples Kruskal-Wallis test

EF	Kruskal-Wallis *p*-value	Q1–Q2 (*p*-value)	Q2–Q3 (*p*-value)	Q2–Q4 (*p*-value)	Q1–Q3 (*p*-value)	Q1–Q4 (*p*-value)	Q3–Q4 (*p*-value)
Age	0.291	0.644	0.708	0.069	0.863	0.346	0.146
WC	0.994	0.987	0.804	0.907	0.834	0.915	0.892
Glucose	** < 0.001**	0.870	0.138	** < 0.001**	0.322	**0.002**	**0.006**
Insulin	0.060	0.322	0.231	0.215	0.950	0.050	**0.013**
HOMA-IR index	**0.037**	0.391	0.556	0.382	0.686	**0.021**	**0.013**
HbA1c	0.704	0.357	0.400	0.311	0.786	0.884	0.869
TC	**0.018**	0.199	0.204	0.120	**0.023**	0.926	**0.004**
TG	0.189	0.889	0.266	0.300	0.468	0.345	**0.029**
HDL	0.223	0.072	0.651	0.800	0.145	**0.044**	0.474
LDL	0.126	0.683	0.033	0.858	0.212	0.784	**0.047**
25(OH)D	**0.019**	0.121	0.475	0.059	**0.034**	**0.002**	0.239

Significant *p*-value < 0.05, *P*-values of statistically significant correlations are highlighted in bold numbers when *p*-value < 0.05

## Discussion

In the current study, the EF thickness of the elderly was mostly above 8 mm, which was the cut-off defined and comes in accordance to other studies conducted in elderly and much higher than the normal EF thickness of younger adults [10–12, 36, 37]. Furthermore, EF was positively correlated with glucose levels in female and total population. The pairwise comparison among EF quartiles showed significant differences in glucose levels, HOMA-IR index, triglycerides, and total cholesterol levels. The values of these variables were mostly significantly higher in the group with the highest EF thickness. In general, the population enrolled in the present study reported a good nutritional status as evidenced by phase angle values that were within the normal range in almost all the older adults, as well as by the levels of Hcy-metabolically related B vitamins which were within the normal reference intervals in most cases. Furthermore, only a small percentage of the population reported sarcopenia, according to EWGSOP2 Consensus [34, 40–42].

Insulin resistance and CVDs are particularly relevant concerns in the elderly population [6–9]; as the world's population ages, IR and CVD are two universal public health problems [6–9]. Even if a causal link has been established linking IR with CVD, the mediating mechanisms are uncertain, and rigorous investigations are needed to fully clarify them [52]. In brief, insulin essentially provides an integrated set of signals that enable nutrient demand and availability to balance [53]. Impaired nutrition contributes to insulin resistance and an increased risk of developing CVD as there are (i) changes in gene expression; (ii) hyperglycemia and dyslipidemia; (iii) activation of oxidative stress and inflammatory response; (iv) endothelial dysfunction and (v) ectopic lipid accumulation, which, exacerbated by obesity, perpetuates metabolic impairment [53].

Over the past decades, EF has been shown to play a significant role in the development of cardiometabolic diseases as its accumulation is associated with atherosclerosis, hypertension, dyslipidemia, IR, and T2DM [9], becoming an important commonality among IR and CVD. However, studies related to EF, IR, and CVD are mostly conducted on patients with cardiometabolic diseases [28–30] and few involve the healthy population, especially the free-living older adults.

Thus, to the best of our knowledge, this is the first nested cross-sectional analysis aimed at exploring the potential correlation between EF and IR-related variables in an older healthy and free-living population.

A meta-analysis by Peters et al. [54] showed that the presence of T2DM in women almost tripled the risk of coronary artery disease (CAD) incidence, while in men was around double. A more recent review by Fourny et al. [55] reported that CVD risk is increased in women with diabetes compared to men and women have a 50% higher risk of CAD in contrast to men. Moreover, according to a systematic review and meta-analysis by Li et al., the amount of EF tissue is significantly higher in patients with type 1 or type 2 diabetes than controls [28]. Christensen et al. consider that the high EF tissue levels in T2DM patients may result in a higher CVD risk, too [56]. Based on a study with coronary disease patients, the adipocyte hypertrophy of EF was significantly associated to increased fasting glucose and decreased insulin levels [57]. This evidence may partially explain the positive correlation between EF and glucose levels correlation in women and not men in this study.

Obesity is a known risk factor for IR and diabetes [56–60]. Iacobellis and Leonetti have shown that EF is a significant factor for obesity-related IR [27]. A study by Chung et al. [61] showed that BMI was positively associated with IR in patients newly diagnosed with T2DM. A study conducted on Iranian women of reproductive age reported that the odds ratios of elevated HOMA-IR index were progressively higher with the increase of WC [62], while Tabata et al. made the same conclusion in Japanese men [63]. As it is noticed in this study insulin and HOMA-IR index were positively correlated with BMI in males, females, and the total population and WC in females and the total population.

In addition, based on literature, the HOMA-IR index has been proposed as a predictor of CVD both in healthy and diseased populations [64]; however, HOMA-IR index cutoffs may differ based on the population. A cross-sectional study on non-obese and non-diabetic participants by Mossmann et al. [64] found a significant association between HOMA-IR above 4.21 and the presence of CAD. According to Bonora et al. [65] that conducted the Verona Diabetes Complications Study, HOMA-IR was recognized as an independent predictor of CVDs in patients with T2DM with a mean age of 64 years. As mentioned before, EF and IR have been related to participants with obesity [29, 35] or populations with diabetes [28] or the presence of CVDs [30]. Moreover, EF thickness may be increased also in patients with type 1 diabetes [66, 67, 68]. Therefore, in the case of this study, statistically significant differences were noticed between lower quartiles of EF thickness and Q4 groups with the highest EF (> 12 mm), which may be contributed to the associations between CVDs and HOMA-IR scores.

Finally, inadequate vitamin D status is a common health problem worldwide [69] and older adults are at high risk for vitamin D deficiency as both production and metabolism change with ageing due to reduced sun exposure and reduced production capacity of the skin [69]. The prevalence of vitamin D insufficiency/deficiency in the present study population was highlighted in more than half population since 64% of older adults reported 25(OH)D levels < 30 ng/ml. However, no information about vitamin supplementation was registered.

Since vitamin D exerts pleiotropic effects in the human body being involved in calcium homeostasis, bone metabolism, and regulation of cardiovascular function, it could be considered a potential common factor between increased EF and IR development. Indeed, several epidemiological studies have shown that impaired vitamin D status was associated with increased risks of different chronic diseases including CVD and diabetes [70]. The results of the present study are following what has already been published by others [71], showing significant differences between 25(OH)D levels and EF quartiles; indeed, as EF thickness increased, 25(OH)D levels decreased. Besides, a negative and significant correlation between EF and vitamin D levels in the female and total population was observed. Studies have shown that vitamin D supplementation could have a potential effect on the expression of various genes in adipocytes [72] and consequently have beneficial effects on epigenetic aging [73]. However, studies specifically on elderly studying the potential association between vitamin D supplementation and EF are not available. Additionally, while studies suggest that vitamin D deficiency may play roles in downstream adverse health consequences, the beneficial effects of vitamin D supplementation on health outcomes remain inconclusive; thus, the causal relationship between vitamin D and health outcomes is still under debate and we could assume that adoption of a vitamin D preventive supplementation strategy for CVD is unlikely to yield any benefit to the general population [69, 70]. Nevertheless, long periods of vitamin D replacement therapy might be particularly useful in older women with increased risk for CVD, recommending long follow-up to demonstrate the potential beneficial effects of vitamin D replacement on epicardial adiposity. It is noteworthy that even if EF has been proven to contribute to inflammation by pro-inflammatory cytokine discharge, and investigations have been performed on vitamin D receptors in different tissues, including the epicardial one [71], no strong evidence of the generalized inverse relationship between vitamin D and EF were obtained and further studies are needed to better elucidate the molecular mechanisms [74]. Finally, in the present study, an inverse and significant correlation was found between 25(OH)D and glucose levels in females and the total population, suggesting how reduced levels of vitamin D could impair pancreatic β-cell functions, thus compromising insulin secretion [75]. However, no significant association was described with insulin levels as well as the HOMA-IR index.

To our knowledge, this is the only study that analyzed the relationship between EF and IR in healthy subjects, while most of the studies have studied the same relationship in populations with diseases [28–30]. Moreover, there is a lack of studies assessing the relationship between CVDs and EF and the use of the HOMA-IR index in exclusively elderly populations. The present study provides a comprehensive and complete evaluation of the cardiometabolic risk assessment as well as of the biochemical and nutritional status of the free-living and healthy older adult. Last, sarcopenia was also assessed by distinguishing between subjects at risk for sarcopenia and subjects diagnosed with sarcopenia by using a hand-grip dynamometer and bioelectrical impedance analysis which are inexpensive, widely available and portable tools recommended for use routinely in hospital practice, in specialist clinical settings as well as in community healthcare, according to the EWGSOP2 standardized procedures and guidelines [34, 40–42].

On the other hand, some study limitations need to be considered. In the first place, the number of participants both for the total population and the EF quartiles is relatively low. Even though, information on potential medication treatment was provided, details about the type of medication and the dose were not present, so it is not known if lipid levels and/or glucose levels were regulated in some of the participants. Another limitation of this study is that it is based on a cross-sectional analysis, so no cause-effect can be concluded. Last, the COVID-19 pandemic restrictions have affected the progress of this research protocol by discouraging participation in the study and contributing to the increase in missing data for those variables that required physical contact to be measured.

## Conclusion

In summary, EF refers to the layer of adipose tissue located around the heart, situated between the outer surface of the heart and the pericardium. In younger individuals, EF is typically minimal and plays a role in providing protection and insulation to the heart. However, as people age, the accumulation of fat becomes more prominent in various regions of the body, including the epicardial area. This age-related increase in EF has sparked interest among researchers and clinicians due to its implications for cardiovascular health. Beyond its local effects, EF has also been implicated in systemic metabolic disturbances; some studies suggest that it might contribute to IR and impaired glucose metabolism, which are commonly observed in the elderly population.

Based on the results of this study and the existing literature, epicardial fat and IR could be related in healthy free-living older adults. Since EF can be a proxy for the determination of overall visceral adiposity, measuring EF thickness can be a useful diagnostic tool and of interest in clinical practice and research. Indeed, measurement of EF thickness could prove useful in age-related risk stratification for CVD, suggesting that echocardiographic measurement of EF thickness should be considered a sensitive and non-invasive measure useful for discriminating older subjects at higher cardiovascular risk. Besides, EF can be easily measured by echocardiography, which is an inexpensive, noninvasive, readily available and reproducible technique.

As the present study is observational and has some limitations mentioned above, we recommend that future studies consider all types of medical therapy and vitamin supplements, including their dose, to clarify whether there are any related health effects to EF and IR. These factors may complement the current evidence provided by this study and allow for further and more in-depth evaluation of a potential association between EF and IR in healthy older adults. Further research with a higher sample size and longitudinal approach should be conducted to arrive at concrete conclusions on the relationship between EF and IR.

## Data Availability

The data presented in this study are available on request from the corresponding author. The data are not yet publicly available due to the plan for further analysis of the same dataset.
